# CIRCULATING MICRORNA PROFILE IN OKINAWAN CENTENARIANS

**DOI:** 10.1093/geroni/igad104.2241

**Published:** 2023-12-21

**Authors:** Sarah Noureddine, Sarah Ashiqueali, Augusto Schneider, Sydney Strader, Richard Allsopp, D Craig Willcox, Bradley Willcox, Michal Masternak

**Affiliations:** University of Central Florida, Oviedo, Florida, United States; University of Central Florida College of Medicine, Orlando, Florida, United States; Universidade Federal de Pelotas, Pelotas, Rio Grande do Sul, Brazil; University of Central Florida, Orlando, Florida, United States; University of Hawaii, Honolulu, Hawaii, United States; Okinawa International University, Ginowan, Okinawa, Japan; Kuakini Medical Center, Honolulu, Hawaii, United States; University of Central Florida College of Medicine, Orlando, Florida, United States

## Abstract

Centenarians are a successful example of aging and extended longevity, exhibiting advanced regulation of biological mechanisms and homeostasis. Since human longevity is a complex field of study that navigates molecular and biological mechanisms that influence aging, we hypothesized that microRNAs, a class of small non-coding RNAs implicated in regulating gene expression at the post-transcriptional level, demonstrate altered expression profiles in circulation across young, middle-aged, and centenarian cohorts. We sequenced circulating microRNAs in Okinawan males and females < 40, 40-80, and >90 years of age accounting for FOXO3A genetic variations (TT and TG alleles) and validated the findings through RT-qPCR. We report 5 microRNAs exclusively upregulated in both male and female centenarians demonstrating predictive functional roles in TGF-β, FoxO, AMPK, Pi3K-Akt, and MAPK signaling pathways. Our findings suggest that these microRNAs upregulated in centenarians may provide novel insight into their enhanced longevity and warrant further exploration into their roles in human aging and longevity.

